# In vitro pH dependent passive transport of ketoprofen and metformin

**DOI:** 10.5599/admet.916

**Published:** 2020-12-09

**Authors:** Alisa Elezović, Amina Marić, Amila Biščević, Jasmina Hadžiabdić, Selma Škrbo, Selma Špirtović-Halilović, Ognjenka Rahić, Edina Vranić, Amar Elezović

**Affiliations:** 1 Department of Pharmaceutical Technology, Faculty of Pharmacy, University of Sarajevo, Zmaja od Bosne 8, 71000 Sarajevo, Bosnia and Herzegovina; 2 Department of Clinical Pharmacy, Faculty of Pharmacy, University of Sarajevo, Zmaja od Bosne 8, 71000 Sarajevo; 3 Department of Pharmaceutical Chemistry, Faculty of Pharmacy, University of Sarajevo, Zmaja od Bosne 8, 71000 Sarajevo, Bosnia and Herzegovina; 4 Control Laboratory of the Agency for Medicinal Products and Medical Devices, Titova 9, 71000 Sarajevo

**Keywords:** dynamic diffusion cell model, artificial membrane, in vitro permeation, BCS II, BCS III

## Abstract

The kinetics of passive transport of ketoprofen and metformin, as model substances for high and low permeability, respectively, across the artificial membrane under the influence of the pH of donor solution was investigated. There was an upward trend in the apparent permeation coefficient (*P*_app_) of ketoprofen with the decrease in pH to a value close to p*K*a. At the pH value below p*K*a the permeation coefficient had lower value, due to the higher retention of ketoprofen in the artificial membrane. Metformin is a low permeable compound, and the highest permeation values were recorded at pH 7.4. Two dissociation constants determine that metformin at physiological pH exists as a hydrophilic cationic molecule, i.e. predominantly in ionized form. At pH values below 2.8, metformin mainly exists in diprotonated form, and it was, thus, very poorly permeable. The highest retention, i.e. affinity of both ketoprofen and metformin to the membrane, was at the lowest pH values, which is explained by different mechanisms. At higher pH values of donor compartment the substances showed significantly less affinity to the membrane. The obtained values of apparent permeation coefficients at studied pH values showed good correlation with the obtained experimental values by other in vitro methods.

## Introduction

The oral route is the most preferred route of drug administration. It is considered safe, effective, and readily available, with minimal discomfort to the patient compared to other routes [1]. The absorption of orally administered drugs is a function of its dissolution, solubility and permeability of the substance along the length of the gastrointestinal tract (GIT) [2]. The absorption of active substances varies depending on the pH along the gastrointestinal tract [3]. In general, the absorption of active substances mainly takes place in the small intestine, i.e. in the jejunum which has the largest absorption surface [4].

The Biopharmaceutical Classification System (BCS) categorizes drugs into four classes according to their water solubility and permeability across intestinal membrane. Based on the available data on low solubility and high permeability of ketoprofen, it belongs to class II (BCS II) [5], while metformin as a highly soluble and low permeable substance belongs to BSC class III [6].

Ketoprofen is a non-steroidal anti-inflammatory analgesic. Within the pH of the gastrointestinal tract, ketoprofen is an ionized substance (weak acid). The p*K*a value of ketoprofen is approximately 4.45 at 25 °C [7].

Metformin is often described as an insulin stabilizer because, due to its positive effects on insulin acceptor and tyrosine kinase activity, it leads to a decrease in plasma insulin levels and insulin resistance [8]. Metformin has two different values of the dissociation constant, namely p*K*a 2.8 and 11.5, and as a result of which at physiological pH it exists as a hydrophilic cationic molecule. At pH values below 2.8, it mainly exists in diprotonated form, and thus difficult to permeate. At pH values higher than 11.5 it is found in non-ionized form which is the most favorable form for passive transport across membranes [9], however this pH is not found physiologically.

Models for determining permeability are used in the early stages of drug discovery and development and are a major strategy to prevent drug rejections due to poor pharmacokinetics in the later, more expensive phases of clinical trials [10]. The most reliable way to determine the permeation of active substances is *in vivo* testing of human intestinal perfusion. However, due to simpler, faster and much less expensive performance compared to the *in vivo* tests, *in situ*, *ex vivo* and *in vitro* models are mainly used for permeation assessment [11].

*In vitro* methods are the most commonly used alternative for testing the permeation of active substances [12]. Depending on the biopharmaceutical class of the active substance, they may have a good correlation with the *in vivo* results [13]. Most active substances are absorbed by passive transport, so the application of *in vitro* methods using artificial membranes that mimic passive transport is considered an effective and reproducible method for assessing permeability [12], especially in screening stages of drug discovery.

Absorption, distribution, metabolism, excretion and toxicity are greatly influenced by the charge of the active substances at different pH conditions. Consideration of pH values in relation to other molecular properties is of great importance and has the potential to be used to further improve the efficacy of the application of active substances [14]. The method of permeation testing on artificial membranes, which could reliably predict the passive transport of the active substance as a function of pH, would significantly facilitate the characterization of new candidate substances in the initial stages of drug development.

In this work, the kinetics of passive transport of ketoprofen (BCS II) and metformin (BCS III), as model substances for high and low permeability, respectively, across the artificial membrane under the influence of the pH value of the donor solution was investigated. The results were compared with the available literature data obtained by different permeation methods of these two substances.

## Experimental

### Materials

Deionized water was obtained on Arium 611, Sartorius (Sartorius Lab Instruments GmbH & Co. KG, Göttingen, Germany). The materials used in this experimental work include: disodium hydrogen phosphate dihydrate (Kemika, Zagreb, Croatia), cholesterol (Sarajevolijek, Sarajevo, Bosnia and Herzegovina), potassium dihydrogen phosphate (Semikem, Sarajevo, Bosnia and Herzegovina), disodium hydrogen phosphate (Kemika, Zagreb, Croatia), ketoprofen, working standard (LEK, Slovenia), metformin hydrochloride, reference standard (Merck, Darmstadt, Germany), sodium chloride (Merck, Darmstadt, Germany), n-octanol (Semikem, Sarajevo, Bosnia and Herzegovina), hydrochloric acid (Lach-ner, sro Neratovice, Czech Republic), 85 % phosphoric acid (Mallinckrodt Baker BV, Deventer, The Netherlands). Egg lecithin (Lipoid® E80) was a kind gift from Lipoid GmbH, Ludwigshafen, Germany.

### Methods

In the experimental work, the Instructions for use of the apparatus absorption simulator Sartorius Model SM 16750 (Sartorius Membranfilter GmbH, Germany) and the method of the authors Corti *et al*. [1,15] and our study group [16] were used. Cellulose nitrate filter (mixed cellulose ester), pore size 0.45 μm (Sartorius AG, Göttingen, Germany) was impregnated with 2 mL solution of 2.10 % cholesterol and 1.70 % egg lecithin (Lipoid® E 80) in n-octanol for 10 minutes. The excess impregnation solution was removed by pressing a filter located between the two absorbent papers. The mass of the filter before and after impregnation was determined using an analytical balance (AG 400, Metler Toledo GmbH, Switzerland) from which the percentage of impregnation solution retained on the filter was calculated as filter mass increase.

**Figure 1.**
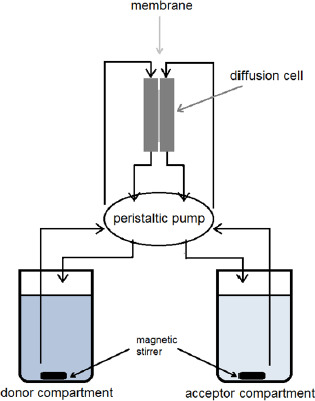
Absorption simulator Sartorius Model SM 16750

The filter prepared in this way was placed in a diffusion cell of absorption simulator connected to the donor and acceptor compartments. The donor compartment contained a solution of 500 μmol L^-1^ of active substance (ketoprofen or metformin) in 100 mL of phosphate buffer of different pH values. The pH values of the buffer in the donor compartment were: 7.4, 6.8, 4.5 and 3.0 or 2.0 for ketoprofen or metformin, respectively. Phosphate buffer of pH 7.4 was present in the acceptor compartment in all experiments. The temperature in both compartments was measured and maintained at 37 ± 0.5 °C. Under the action of the peristaltic pump, the contents of the donor and acceptor compartment were constantly circulating through the diffusion cell on both sides of the membrane (Figure 1).

At 30, 60, 90 and 120 minutes, sampling was performed from the acceptor compartment and the concentration of active substances (ketoprofen or metformin) was determined spectrophotometrically. Spectrophotometric measurements were performed on UV-VIS spectrophotometer UV-1601 (Shimadzu, Kyoto, Japan). The method was validated prior to the permeation experiments according to ICH guideline Q2-R1 [17].

After reading the results on the spectrophotometer, the samples were returned to the acceptor compartment. At the end (120 minutes), the samples were also taken from donor compartment, and concentration of ketoprofen or metformin was determined spectrophotometrically. This data was used to calculate the recovery value (*R*). Each experiment was performed in triplicate.

From the samples taken from the acceptor compartment, the apparent permeability coefficients of ketoprofen and metformin were calculated based on Equation 1:


(1)

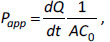



where *P*_app_ - apparent permeation coefficient, cm s^-1^; d*Q*/d*t* - mass of the substance that passed through the membrane per unit time, mg s^-1^; *A* - effective surface of the artificial membrane through which the permeation was examined, cm^2^; *C*_0_ - initial concentration of active substance in donor solution, mg mL^-1^.

Recovery of ketoprofen and metformin (as a percentage of the substance not retained in the membrane) was determined according to Equation 2:


(2)

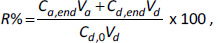



where *C*_a, end_ and *C*_d, end_ - concentration of test substance measured at the end of the experiment in acceptor and donor solution, mg mL^-1^; *C*_d,0_ - initial concentration of test substance in donor solution, mg mL^-1^; *V*_a_ and *V*_d_ - volumes of acceptor and donor solution, mL.

## Results and discussion

Permeations of ketoprofen and metformin were monitored in this experimental work at different pH values of the donor compartment, while the pH of the acceptor compartment was 7.4 in all cases. In this way, it was possible to examine the permeation of the substances from the donor compartment at different pH values, which simulated GIT conditions across an artificial membrane embedded in a diffusion cell in the acceptor compartment that reflected the pH conditions of the systemic circulation.

A membrane filter with pore size of 0.45 μm was used in this work. As we mentioned in the earlier work [16] the pore size of the membrane filter does not affect the permeability of active substances across the artificial membrane, since in Corti *et al*. [1] there was no clear causal relationship between *P*_app_ and pore sizes established.

According to the Instructions for use of the apparatus (Sartorius Model SM 16750), the increase in filter weight after impregnation should be 100 ± 5 %. The mean value of the percentage of impregnation of the membrane, i.e. the average increase in filter mass was 158.67 ± 5.59 %. These values are similar to the values of experimental studies conducted by Corti *et al*. [1] and our study group [16]. According to both studies, the structure of a membrane filter that was not originally intended for use in this experiment is responsible for the larger increase in filter mass after impregnation.

### Permeation of ketoprofen dependent on the pH of the donor solution

**Figure 2.**
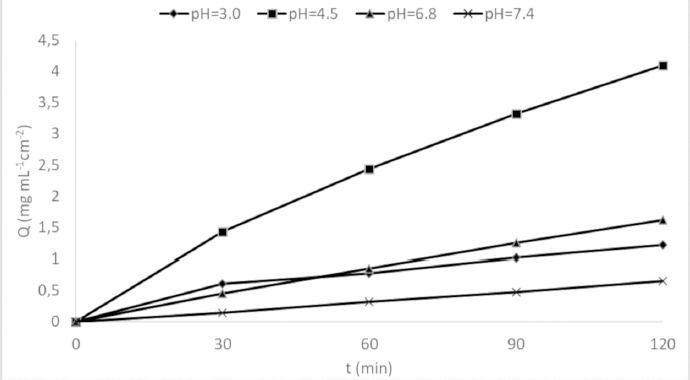
Overall permeated amount of ketoprofen through the artificial membrane at different pH values of donor compartment.

The ketoprofen permeation profiles' parameters at different pH of the donor compartment are presented in Table 1 and Figure 2.

Large values of the apparent permeation coefficients confirm that it is a highly permeable substance. The low standard deviation of the apparent permeation coefficients shows good reproducibility of the method for highly permeable substances. The recovery values indicate whether ketoprofen was retained in the membrane, and this retention varied significantly depending on the pH value.

In general, ionized substances are better soluble, and non-ionized ones pass through the cell membrane better. The calculated percentage of non-ionized ketoprofen decreased from 96.61 % for pH = 3.0 to 0.11 % at pH = 7.4 (Table 1). Ketoprofen is a weak acid and its maximum permeation is expected in areas where the pH value is close to the p*K*a of the substance, which in the case of ketoprofen is 4.45, where there is 47.17 % non-ionized ketoprofen. These obtained calculated values of the proportions of ionized and non-ionized species explain the values of the apparent permeation coefficients and recovery factors at individual pH, discussed below.

It can be seen from Figure 2 that the equilibrium permeation concentrations were not established at any pH during the 120-minute test period.

In general the values of *P*_app_ in all pH media were high (Table 1). Such results indicate high permeability of ketoprofen, which is in line with the literature data showing that ketoprofen belongs to BCS class II.

The recovery value increased with increasing pH (Table 1), and for pH = 7.4 the recovery value was 97.95 %. Such a high value indicates that a very small percentage of the substance was retained by the membrane. The recovery value decreased with decreasing pH, and the lowest percentage of the recovered substance was measured at pH = 3 and it was R = 51.93 %. In case of weak acids, such as ketoprofen, the proportion of non-ionized substance is increased at pH values below p*K*a, i.e. pH of 3.0 in this case. The increased portion of non-ionized substance makes this substance more lipophilic and thus has a higher affinity for the lipophilic membrane.

When analysing the obtained values of apparent permeation coefficients from Table 1, it can be noticed that unlike recovery values, *P*_app_ did not have a linear growth with pH values. The lowest mean value of the apparent permeation coefficient (*P*_app_ = 6.95 ± 0.51 × 10^-5^ cm s^-1^) was at pH = 7.4. Regardless of the fact that the recovery value at this pH shows the highest percentage, and *P*_app_ is the lowest, such results are consistent with the results obtained in the original method. In the original *P*_app_ method, the value for ketoprofen at pH = 7.4 was 4.27 ± 0.08 × 10^-5^ cm s^-1^ and *R* = 99.1 % [15]. The apparent permeation coefficient showed the highest values at pH = 4.5, which was to be expected given that the maximum permeation of ketoprofen is expected at pH values that are closest to the p*K*a value of the substance, which for ketoprofen has a value of 4.45. This is due to the fact that at this pH value there is a higher ratio of non-ionized substance, which permeates, compared to higher pH values, and, on the other hand, there is less retention in the membrane compared to lower pH value that is bellow p*K*a value, probably due to the larger portion of the ionized form. The apparent permeation coefficient was 41.6±1.67 × 10^-5^ cm s^-1^. This result confirmed the high permeability of ketoprofen, and is in line with the fact that ketoprofen is a weak acid, the absorption of which is expected at a weakly acidic to neutral pH values. Measurement with phosphate buffer in the donor compartment having a pH of 6.8 resulted in a *P*_app_ value of 16.8±1.1 × 10^-5^ cm s^-1^. These values correlate with all the above facts related to the permeation properties of ketoprofen. At pH = 3.0, the mean *P*_app_ value obtained was 12.9±0.28 × 10^-5^ cm s^-1^. These results were expected, and it is assumed that if there was no increased affinity of the substance and the membrane, it would be even higher at this pH value.

Numerous methods for testing absorption/permeation have been described in the literature. Table 2 shows the literature accounts of experimentally obtained values of other methods for testing the permeation of ketoprofen at certain pH values, as well as the literature values of the *in silico* simulated dose fraction absorbed in humans (*F*a), and log *D*. The correlations between the obtained results and these literature data were examined. In general, this type of testing referring to pH dependence is scarce and lacking.

Due to the lack of experimental data on the permeation coefficients of ketoprofen at the exact same pH values, the values at approximate pH were taken for certain parameters, as indicated in the Legend of Table 2.

Dose fraction absorbed in humans of ketoprofen is 90 %, and it is mainly absorbed in the small intestine [5]. However, due to the lack of pH dependent data on its *in vivo* absorption, the obtained results cannot be compared or correlated. The results were compared with the available results of *in silico* simulated fraction of the dose absorbed in humans at different pH values and gave the correlation factor of *R*^2^ = 0.9239.

The comparisons of the results of *in situ* and *ex vivo* models [20] were possible only at two pH values, namely pH = 6.8 and pH = 7.4, so it was not purposeful to calculate the correlation coefficient. However, the trend of increasing permeation with decreasing pH of the environment in both cases was evident (Table 2). The same trend was present for the BML-PAMPA assay and log *D* results, even taking into consideration that the pH was different near p*K*a of ketoprofen in the two experiments.

Comparison of the obtained results with the results of Corti *et al*. [15] and our earlier results [16] was not possible due to the lack of data on apparent permeation coefficients for all pH values.

### Permeation of metformin dependent on the pH of the donor solution

**Figure 3.**
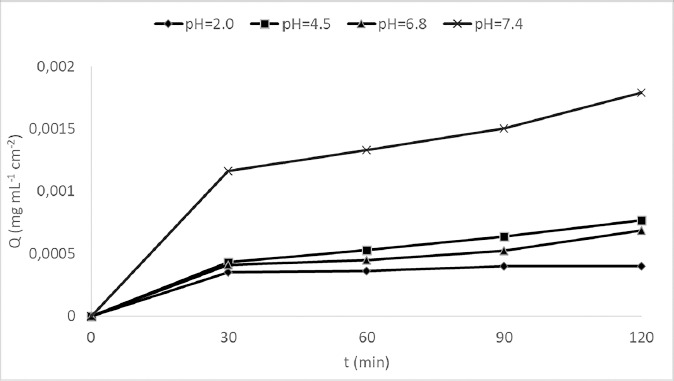
Overall permeated amount of metformin across the artificial membrane at different pH values of donor compartment.

The metformin permeation profiles' parameters at different pH of the donor compartment are presented in Table 3 and Figure 3.

The p*K*a value of 11.5 makes metformin a strong base found in non-ionized form in the percentage of only 0.008 % at pH 7.4. Given the physicochemical properties of metformin (p*K*a values, log *P*) that determine its high solubility and poor permeability, metformin will prevail at physiological pH in ionized form, which will favor its solubility, but will not favor passive transport. Due to its properties, metformin is mainly absorbed *in vivo* by active transport via number of transporters located in intestinal membranes [22]. Otherwise, for drugs belonging to BCS class III, it would be possible to establish an *in vivo - in vitro* correlation if the drug is highly soluble (such as metformin), if it is not subject to the first pass effect and is not metabolized (such as metformin) and if it is not transported by carriers. Since it does not meet the third criterion, it is not possible to establish an *in vitro - in vivo* correlation for metformin, but it is possible to examine the extent to which passive transport is involved in its permeation and the effects of pH on this type of permeation.

At pH 2.0 (which corresponds to the gastric pH) most of metformin (86.32 %) is in diprotonated form, i.e. it is in the form of a double positively charged ion. In the protonated form, positively charged metformin at this pH value is present at 13.68 %, while the non-ionized form is represented by only 4.33 × 10^-9^ %. These data point to the fact that metformin at the pH value of the stomach is very difficult to passively permeate through physiological membranes, which the obtained results confirm.

Table 3 shows that the *P*_app_ value was 5.53 × 10^-8^ cm s^-1^, very low due to the low permeability of the substance at pH 2.0. Metformin is at gastric pH, a highly ionized molecule and as such cannot passively permeate through physiological membranes. The recovery value was 85.31 %, indicating a higher retention of metformin in the artificial membrane than at other pH values of the donor compartment, probably due to the affinity of metformin for membrane components made of cellulose nitrate and phospholipids from the impregnation solution.

At pH 4.5, the share of the diprotonated form of metformin is 1.95 %, while the single positively charged form dominates (98.04 %). Non-ionized form of the molecule is present in a percentage of 9.8 x 10^-6^ %. Although present in extremely low percentage, the non-ionized form of metformin is still insufficient for unobstructed passive permeation, and the *P*_app_ value was 9.79 ×10^-8^ cm/s at this pH. The pH value of 4.5 is present at the level of the duodenum from where *in vivo* metformin absorption will start to take place.

At pH values of 6.8, metformin is found in the non-ionized form in portion of 0.0019 %. Passive permeation in this case is more pronounced than at lower pH values. Generally, *in vivo* metformin absorption mainly takes place from GIT regions where the pH value is 6.8. However, *in vivo* absorption is aided by a number of protein carriers and transporters located along the length of the intestinal mucosa that facilitate and enable cellular uptake of metformin from the apical to the basolateral membrane of enterocytes. Therefore, an *in vitro* model based exclusively on passive transport could not adequately map events at physiological pH 6.8, but only the passive component of membrane transport. Since the non-ionized content is lower than at pH 7.4, the permeation rate is lower at this pH of the environment. The mean *P*_app_ value shown in Table 3 was 9.83 ×10^-8^ cm s^-1^.

At pH 7.4 metformin is present in the non-ionized form 0.008 %. Although the concentration of the uncharged form is extremely low, it is the highest at the studied pH values and as such shows the best permeation rate and extent through the artificial membrane.

The pH value of the donor compartment was the furthest from the lower p*K*a value for metformin (2.8), which favored its existence in non-ionized form, and enabled its passive permeation across the artificial membrane. From the results shown in Table 3, it can be seen that the mean *P*_app_ at pH 7.4 was 2.98 × 10^-7^ cm s^-1^. Although this permeation rate is low compared to substances that show good permeability, this is the highest value of permeation obtained in this experimental work due to the fact that at these pH values the highest percentage of non-ionized form is present compared to other studied pH values. Thus, metformin is a low permeable compound whose absorption is more favored by alkaline pH values (Figure 3). The recovery value of *R* = 96.19 % (Table 3) indicates that metformin mostly uninterruptedly permeated.

In summary, metformin is highly ionized throughout the length of the GIT, which explains its low permeability by passive transport. It also explains the fact that metformin is in BCS class III (high solubility - low permeability). Its permeability, as mentioned earlier, is significantly higher *in vivo*, since it is suggested to be absorbed by active transport through supported carriers [22].

In Figure 3, it can be seen that as the pH value increases, the slopes of the metformin permeation profile increase, so the highest *P*_app_ values were observed at pH 7.4. At this pH value, metformin has good solubility, low lipophilicity, and the highest concentration of non-ionized substance compared to the other studied pH values, thus it more easily permeates into the acceptor compartment with very small percentage of retention in the artificial membrane. The log *D* value for metformin is -1.43, which indicates low solubility in lipids, and thus low affinity for the membrane, which is lipid in nature. As can be seen from Figure 3, after the initial jump in the concentration of permeated metformin after 30 minutes at pH 2.0, almost a plateau is reached, which can be explained in that the saturation of metformin in the membrane is reached and diprotonated molecules repel each other impeding further permeation through the membrane. This is similar, although less pronounced, at higher pH values.

The behavior of metformin at pH values of 6.8 and 4.5 corresponding to the small intestine was shown to be very similar (Figure 3). Even though low permeation rates were observed at both pH values, permeation of metformin at pH 4.5 physiologically present in the duodenum showed a slight, although insignificant, advantage.

In humans, metformin absorption is incomplete and bioavailability shows interindividual variability at 55 ± 16 %. Absorption ceases 6-10 hours after administration, regardless of the amount of metformin administered, which is approximately the time of passage of the drug through the stomach and small intestine [27]. Human data are lacking in pH dependent transport across the GIT membranes, and thus cannot be further discussed.

Lassoued *et al*. [23] in their comparative study of permeation of metformin by the Sartorius SM 16750 absorption simulator model and inverted sac technique showed a good correlation in the determination of metformin permeability.

The results of the study conducted by Song *et al.* [24] concluded that metformin is transported via saturable carriers. The results were not correlated with dynamic diffusion cell model due to the difference in the used methods.

However, in the study conducted by Nicklin *et al*. using the Caco-2 model it was concluded that metformin is transported in the Caco-2 monolayer by passive, unsaturable transport [25]. The hydrophilicity and molecular weight of metformin determined the *P*_app_ value of 5.5 × 10^-6^ cm s^-1^, which is comparable with the results of other hydrophilic high molecular weight compounds tested by the Caco-2 model [28], but also with the results obtained in this experimental work at the same pH values (Table 4). On the other hand, in the Caco-2 cell model study conducted by Proctor *et al*. [26] the transport and absorption of metformin took place via saturable transport mechanisms. The relative contribution of transcellular and paracellular transport of metformin at a concentration of 0.05 mM was estimated at 9 % and 91 %, respectively [26].

Log *D* values at the studied pH were below 0.5 which implies poor permeability of metformin within the physiological pH range prevailing at the GIT level, which is in line with the obtained results. However, the correlation of the results with the available log *D* values was very poor (*R*^2^ = 0.63).

## Conclusions

Ketoprofen is a highly permeable acid, while metformin is dibasic substance with low permeability. pH dependent passive transport through an artificial biomimetic membrane of both substances could be explained by their ionization properties at studied pH values of donor compartments. Log *D* values of ketoprofen correlated well, while those of metformin did not. The literature accounts of pH dependent permeation/absorption are scarce. However, when considering those available, the results correlated well with the other *in vitro* studies.

The dynamic diffusion cell model has proven to be an efficient, reproducible and simple method for predicting the permeation of ketoprofen. This model examines passive transport across the membrane, and for substances that are absorbed by other forms of transport, such as metformin, this method can be used to predict only its passive transport component.

The dynamic diffusion cell model is a good foundation for future research as a valuable resources-saving screening tool for the new drug candidates at the beginning stages of their development, as well as for known substances permeation elucidation, especially considering its potential for testing at wide range of pH conditions, which is very difficult by other methods.

## Figures and Tables

**Table 1.** Mean values (n = 3) of the apparent permeation coefficients (*P*_app_) of ketoprofen, the recovery of the substance not retained by the membrane (*R*) and percentage of calculated (non)ionized species (HA/A^-^) at different pH values of donor compartment.

pH	*P*_app_ (× 10^-5^ cm s^-1^) ± SD	*R*(%) ± SD	HA/A^-^ (%)
**7.4**	6.95 ± 0.51	97.95 ± 2.42	0.11 / 99.89
**6.8**	16.8 ± 1.10	87.83 ± 2.77	0.44 / 99.56
**4.5**	41.6 ± 1.67	83.52 ± 1.68	47.17 / 52.83
**3.0**	12.9 ± 0.28	51.91 ± 1.79	96.61 / 3.39

**Table 2.** Experimentally obtained apparent permeation coefficients of ketoprofen (cm s^-1^) at different pH values and literature data obtained by other methods.

pH	*P*_app_ ± SD (×10^-5^)	*F*a %^a^	*P*_app_ ± SD (×10^-5^)^b^	*P*_app_ ± SD (×10^-5^)^c^	*P*_eff_ ± SD (×10^-5^)^d^	log *D*^e^
**3.0**	12.9 ± 0.28	/	/	/	/	/
**4.5**	41.6 ± 1.67	99.9	6.02 ± 2.4**	/	/	1.8**
**6.8**	16.8 ± 1.1	100	1.86 ± 1.5***	26.0 ± 13.4***	29.2 ± 0.7***	0.8
**7.4**	6.95 ± 0.5	100	0.24 ± 0.7	15.8 ± .24	23.5±0.7	0.1

^a^Values of simulated *F*a (dose fraction absorbed in humans) [18]

^b^BML-PAMPA (biomimetic lipid parallel artificial membrane permeability assay) *P*_app_ values [19]

^c^*P*_app_ values obtained by Ussing chambers' model [20]

^d^SPIP *P*_eff_ (effective permeation coefficient) values [20]

^e^Log *D* (distribution coefficient) values [20,21]

* Values at pH = 2

**Values at pH = 5.5

***Values at pH = 6.5

**Table 3.** Mean values (n = 3) of the apparent permeation coefficients (*P*_app_) of metformin, the recovery of the substance not retained by the membrane (*R*) and percentage of calculated (non)ionized species (BH_2_^2+^/BH^+^/B) at different pH values of donor compartment.

pH	*P*_app_ (× 10^-8^ cm s^-1^) ± SD	*R*(%) ± SD	BH_2_^2+^/BH^+^/B (%)
**7.4**	29.82 ± 1.095	96.19 ± 4.92	0.0025 / 99.99 / 0.008
**6.8**	9.83 ± 0.199	90.00 ± 2.73	0.010 / 99.99 / 0.002
**4.5**	9.79 ± 0.234	90.37 ± 3.20	1.96 / 98.04 / 9.8 × 10^-6^
**2.0**	5.53 ± 0.556	85.31 ± 4.45	86.32 / 13.68 / 4.33 × 10^-9^

**Table 4.** Experimentally obtained apparent permeation coefficients (cm s^-1^) of metformin at different pH values and literature data obtained by other methods.

pH	*P*_app_ ± SD (×10^-6^)	*P*_app_± SD^a^ (×10^-6^)	*P*_app_ ± SD^a^ (×10^-6^)	*P*_app_ ± SD^b^ (×10^-6^)	*P*_app_ ± SD^b^ (×10^-6^)	*P*_eff_ ± SD^c^ (×10^-6^)	log *D*^d^	*P*_app_^d^ (×10^-6^)	*P*_app_ ± SD^e^ (×10^-6^)
**2.0**	0.055±0.006	/	/	/	/	/	/	/	/
**4.5**	0.098±0.002	/	/	/	/	45.1±10.8 (duodenum)	-1.41	/	/
**6.8**	0.098±0.002	V 0.74±0.15	S 0.61±0.1	/	/	32.6±7.3 (jejunum) 29.6 ± 3.6 (ileum)	-1.30 -1.31	/	0.50±0.057
**7.4**	0.298±0.011	/	/	A 8.6±1.4	B 10.9±1.9	/	-1.23	5.5	/

^a^Values *P*_app_ obtained by inverted sac and Sartorius model [23]

^b^Values *P*_app_ obtained on the same Sartorius model with the same experimental conditions with impregnation solution variation [16]

^c^Values *P*_app_ obtained on isolated duodenum, jejunum and ileum segments in Caco-2 cell model [24]

^d^Values log *D* and *P*_app_ obtained by Caco-2 cell model [25]

^e^Values *P*_app_ obtained by Caco-2 cell model [26]
